# Supplementary material for: Mapping lung cancer epithelial-mesenchymal transition states and trajectories with single-cell resolution
Source: Nat Commun. 2019 Dec 6;10:5587. doi: 10.1038/s41467-019-13441-6 (PMC6898514; doi:10.1038/s41467-019-13441-6)
Supplement: Supplementary file 1 — Supplementary Information [file 41467_2019_13441_MOESM1_ESM.pdf]

Supplementary Information

Mapping Lung Cancer Epithelial-Mesenchymal Transition States and Trajectories with Single-Cell Resolution

Karacosta et al.

Antibody Target	Clone	Vendor	Metal	Mass	Final Concentration (ug/mL)
CD45	H130	Biolegend	Y	89	1
FAP	F11-24	eBioscience	Ln	113	1
CD44	IM7	Biolegend	Ln	115	1
cleaved Caspase 3*	C92-605	BD	Nd	142	1
phospho-Src	K98-37	BD	Nd	144	1
phospho-EGFR	D745	CST	Nd	145	2
EGFR	D38B1	CST	Nd	146	1
TROP2	77220	R&D	Nd	148	3
Oct 3/4	O50-808	BD	Nd	150	4
Notch3	MHN3-21	Biolegend	Eu	151	1
Cytokeratin 8	SP102	Abcam	Sm	152	1
PD-L1	29E.2A3	Biolegend	Eu	153	2
MUC1	SPM492	Abcam	Sm	154	2
RUNX1	1C5B16	Biolegend	Gd	155	2
Snail	C15D3	CST	Gd	156	4
E-Cadherin	67A4	Biolegend	Gd	158	2
Nanog	polyclonal	CST	Tb	159	2
phospho-H3	HTA28	Biolegend	Gd	160	2
CD24	ML5	Biolegend	Dy	161	4
phospho-SMAD2/3	D27F4	CST	Dy	162	2
phospho-NFKb	K10-895.12.50	BD	Dy	163	2
phospho-S6	N7-548	BD	Dy	164	2
phopsho-Rb	J112-906	BD	Ho	165	0.5
Cytokeratin 7	SP52	Abcam	Er	167	0.5
Twist	polyclonal	Bioss	Er	168	4
non phospho-b-catenin	D13A1	CST	Er	170	1
CD31	WM59	Biolegend	Yb	171	0.5
Slug	C19G7	CST	Yb	172	4
CD104	58XB4	Fluidigm	Yb	173	2
Vimentin	D21H3	CST	Yb	174	2
phospho-AMPK	40H9	CST	Lu	175	2

* Replaced in certain runs with cleaved PARP antibody (Clone: F21-852, Vendor: BD, Pr 141, 1ug/mL)

Supplementary Table 1. Mass cytometry EMT-MET antibody panel. Related to Figure 2. Antibodies in grey boxes were included in mass cytometry runs of NSCLC clinical specimens to separate immune (CD45), stromal (FAP) and endothelial (CD31) cell populations from tumor cells (see Methods for additional information).

PCA1	PCA2	PCA3
pRb	**CD44**	**Twist**
pEGFR	**Vimentin**	Cytokeratin7
Nanog	**ECadherin**	pEGFR
Cytokeratin8	pAMPK	pS6
pS6	Oct3/4	
pNFKB	TROP2	
**CD24**	Slug	
pSmad2/3	**MUC1**	
**ECadherin**		

Supplementary Table 2. Statistically significant markers resulting from principal component analysis (PCA) of mass cytometry data. Related to Figure 3. In bold are the 6 EMT markers selected among all the markers for our CCAST analysis. E-Cadherin, Vimentin, CD44, CD24, MUC1 and Twist were among the most statistically significant markers that correlated independently with the top 3 principal components which explained about 50% variability in the data. (p-value <0.001, global test of independence constructed by means of the conditional distribution of linear statistics in the permutation test framework) See Methods for additional information.

MET vs. M

	logFoldChange	Statistic	Parameter	p-value	Adj p-value
**TROP2**	0.63519163	-42.53892	19202.7	0	0
**Cytokeratin 8**	0.46141363	-24.83361	17692.38	7.56E-134	2.34E-132
**Nanog**	0.29493824	-23.04585	17203.75	1.31E-118	4.06E-117
**pS6**	0.26344908	-23.33111	17714.73	9.12E-116	2.83E-114
**pH3**	0.34189283	-20.27695	17209.39	2.33E-90	7.23E-89
**pSrc**	0.28489028	-18.71282	16187.7	2.54E-77	7.89E-76
**c-Caspase 3**	0.21345468	-15.27348	16260.63	1.30E-54	4.03E-53
**pNFKb**	0.28484486	-15.61989	16657.43	2.65E-52	8.21E-51
**b-catenin(non-p)**	0.21076742	-13.2264	15771.97	1.01E-39	3.12E-38
**pEGFR**	0.20388849	-13.20525	17158.95	1.28E-39	3.96E-38
**Oct3/4**	0.16019264	-8.606327	16799.11	8.20E-18	2.54E-16
**pSMAD2/3**	0.13855097	-8.301525	16363.19	1.11E-16	3.43E-15
**PD-L1**	-0.08843838	6.818008	17442.74	9.53E-12	2.96E-10
**pAMPK**	0.13018351	-6.018404	16542.6	1.80E-09	5.58E-08
**RUNX1**	0.07195712	-5.128097	16138.89	2.96E-07	9.18E-06
**pRb**	0.0454928	-3.660522	16386.71	0.000252	0.007827
**EGFR**	0.05858275	-3.555853	16816.33	0.000378	0.011711
**Snail**	0.03501546	-2.702721	16499.43	0.006884	0.213419
**Cytokeratin 7**	-0.0553096	2.667408	16368.39	0.007651	0.237194
**Slug**	0.01389564	-1.227157	16363.12	0.219781	1
**Notch3**	0.01144468	-1.031609	16579.16	0.30227	1
**CD104**	0.01225016	-0.879444	16692.62	0.379173	1

Supplementary Table 3. TTEST analysis between M and MET states. Markers are ranked according to their p-value. The 6 clustering markers used in CCAST (E-Cadherin, Vimentin, CD44, CD24, MUC1 and Twist) were not incorporated for this analysis. Statistics: t-statistics used for calculating the p-values, Parameter: degree of freedom which is the only parameter for the t-distribution similar to mean and variance for the normal distribution, Adjusted p-value: p-values based on Bonferoni corrections.

Case No.	Description	Grade (Differentiation)	Size (cm)	Mutations	Smoking History
1	NSCLC, Adenocarcinoma	Well	4.7	EGFR (exon 19 deletion)	Never
1	NSCLC, Adenocarcinoma	Moderate	4	EGFR (p.Leu858Arg)	Former
2	NSCLC, Adenocarcinoma	Poor	2.5	EGFR (exon 19 deletion) TP53 (p.Asp259Val)	Never
3	NSCLC, Adenocarcinoma	Poor	5	TP53 (p.Arg248Leu)	Former
4	NSCLC, Adenocarcinoma	Moderate/Poor	3.5	KRAS (p.Gly12Ala)	Former

Supplementary Table 4. Clinical data of the 5 patient specimens analyzed with mass cytometry

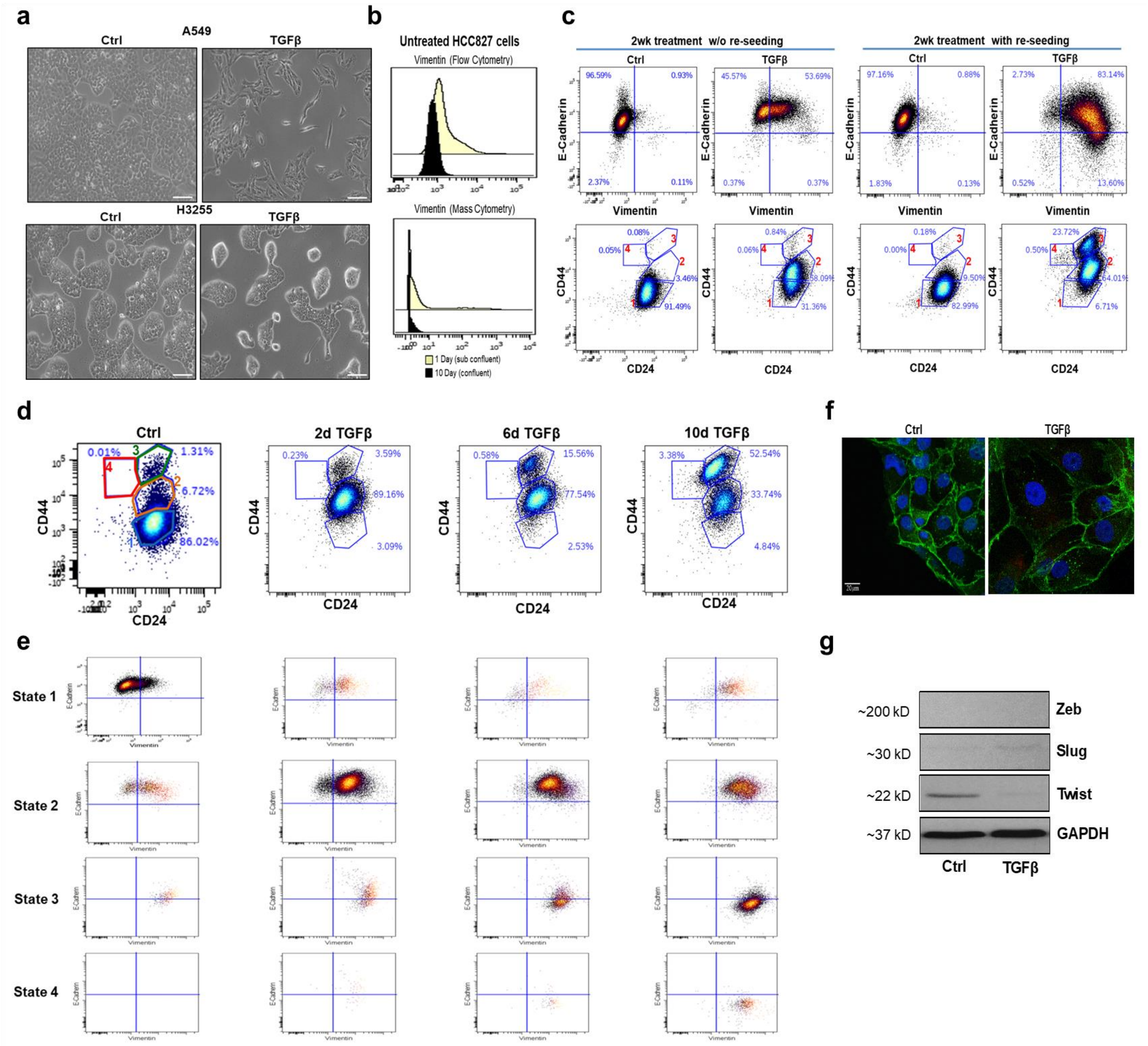

Supplementary Figure 1. Optimization of TGFβ-induced EMT in NSCLC cell lines and identification of canonical EMT states via flow cytometry. Related to Figure 1. **(a)** Representative images of A549 and H3255 cells treated with TGFβ (5ng/mL) for 1 and 2 weeks respectively. Magnification 10x, scale bar 200 μm. **(b)** Vimentin levels in untreated HCC827 cells that were in either sub confluent (yellow color, seeded and collected after 1 day) or confluent (black color, seeded and collected after 10 days) cell culture conditions. Top graph, flow cytometry analysis, bottom graph, mass cytometry analysis of an independent biological replicate experiment. **(c)** E-Cadherin/Vimentin and CD44/CD24 flow cytometry plots of HCC827 cells treated with TGFβ for 2 weeks in non-re-seeding (left). and re-seeding cell culture conditions (right). Shown are the respective 4 canonical EMT states and % of cells per gated area. **(d)** CD44/CD24 flow cytometry plots shown in Figure 1A, with the respective 4 gated states and % of cells per gated area**.** **(e)** Gated states 1 through 4, shown independently towards their E-Cadherin/Vimentin phenotype expression. **(f)** Representative confocal images of HCC827 cells treated with TGFβ (5ng/mL) for 6 days and stained for E-Cadherin (green). Note the significantly larger cell size of cells treated with TGFβ compared to ctrl cells, while still retaining E-Cadherin expression at tight junctions. Scale bar 20

μm. **(g)** Immunoblots of EMT transcription factors Zeb, Slug and Twist in HCC827 cells treated with TGFβ for 2 weeks. Source data are provided as a Source Data file.

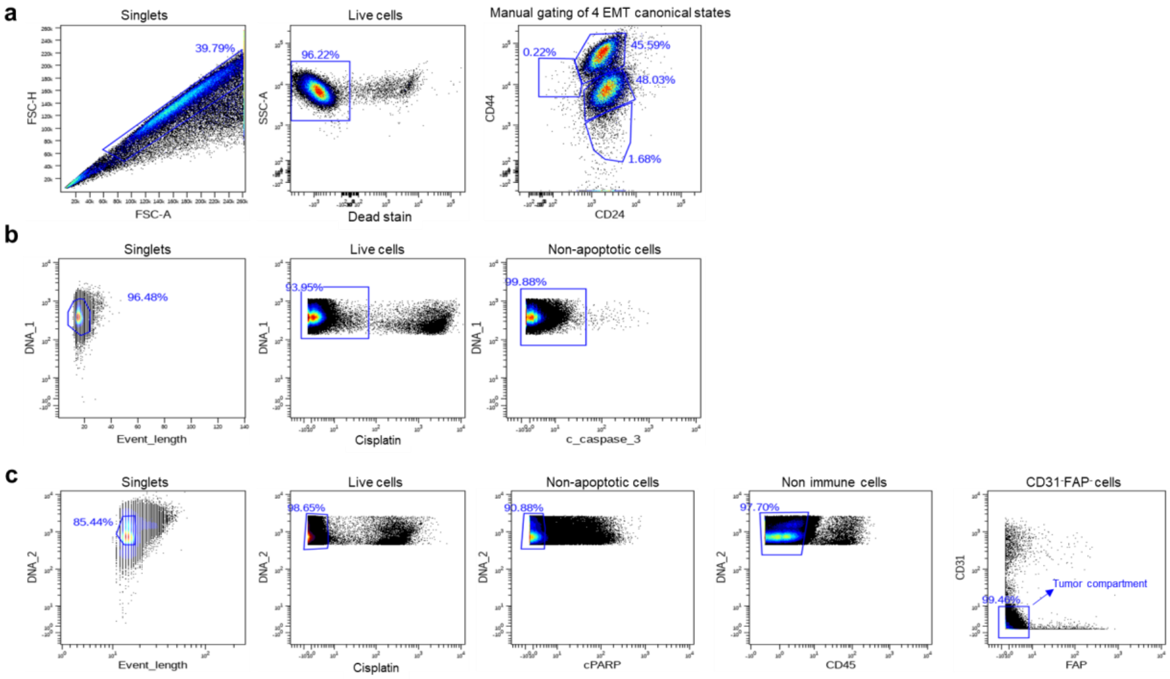

Supplementary Figure 2. Gating strategies and hierarchy (left to right) related to Figures 1, 2, 5, 6 and Supplementary Figure 1. **(a)** Gating strategy for HCC827 cells analyzed with flow cytometry. Shown here, a biological replicate of a 2 week TGFβ treatment with re-seeding conditions (Fig. 1 and Supplementary Fig. 1). **(b)** Gating strategy for HCC827 control cells analyzed with mass cytometry (Fig. 2). Following de-barcoding, hand-gating of DNA and cell length parameters were used to separate single cells from debris and doublets. Then, non-viable (cisplatin positive), and apoptotic (cleaved caspase 3 or PARP positive) cell events were removed. The same gating strategy was followed for analyzing A549 and H3255 cells (Fig. 5) with mass cytometry. **(c)** Gating strategy for lung cancer clinical specimens (Fig. 6). After removing doublets, debris and non-viable and apoptotic cells, we further separated tumor cells from immune (CD45+), endothelial (CD31+) and fibroblast cell populations (FAP+). Shown here is Case No. 2.

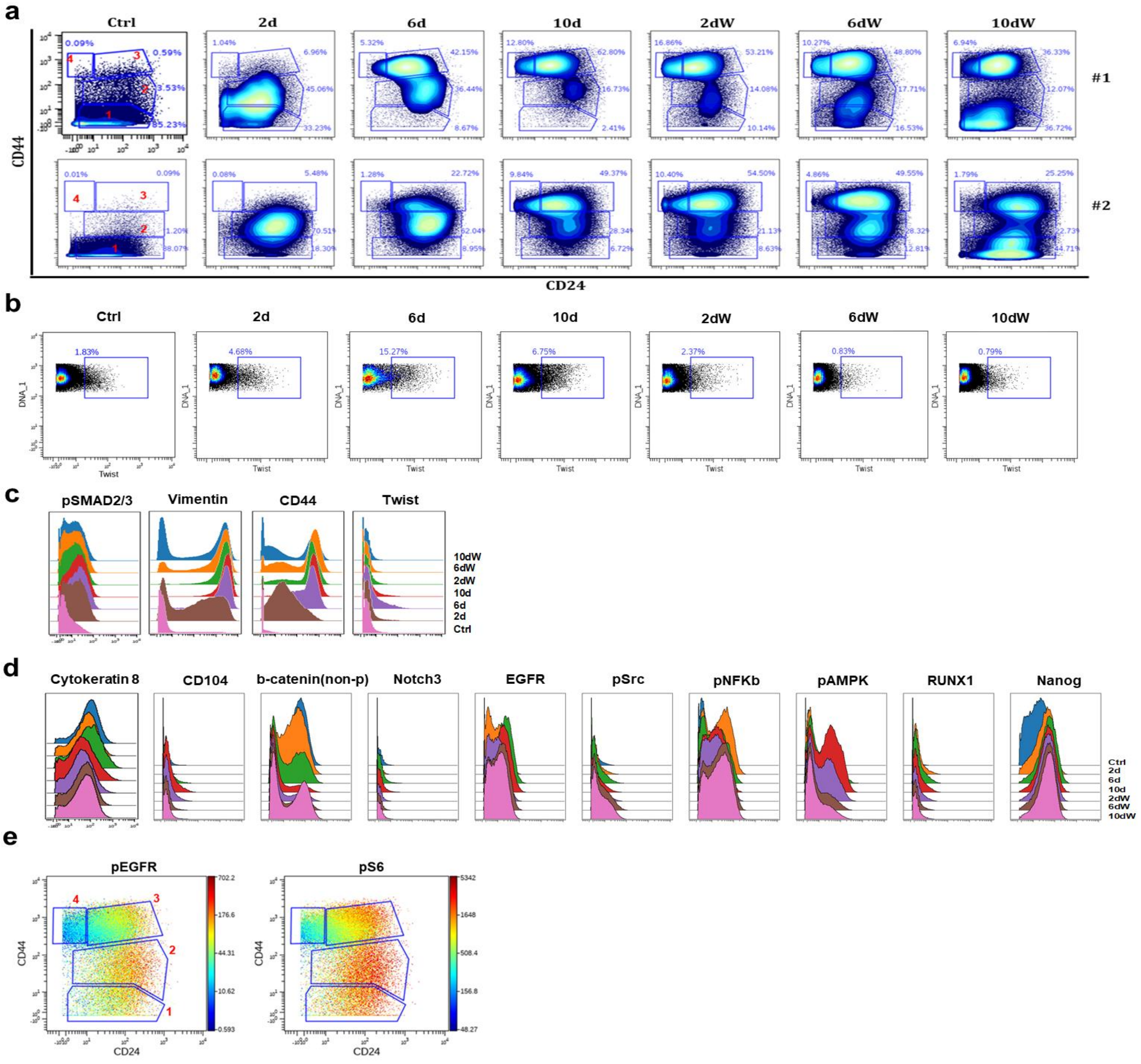

Supplementary Figure 3. Time-course analysis of EMT and MET with mass cytometry. Related to Figure 2**. (a)** Mass cytometry measurements of CD44/CD24 expression changes confirm the existence of the 4 canonical EMT states observed in HCC827 cells with flow cytometry analysis in two independent biological TGFβ time-course replicates. Shown also are % of cells in each of the 4 states and how these change with time. **(b)** Gating Twist positive cells in HCC827 time-point samples (depicted in Figure 3) shows the transitional increase in numbers during EMT (4 and 6d TGFβ) and subsequent decrease prior time-points in which the majority of cells become most mesenchymal (10d TGFβ and 2dW). **(c)** Reverse histogram overlay (time-point representation) to show basal levels of pSMAD2/3, Vimentin, CD44 and Twist**. (d)** Remaining cellular markers measured with mass cytometry in the HCC827 TGFβ time-course depicted in Figure 2. **(e)** State 4 ($CD44^{hi}/CD24^{lo}$) cells express low pEGFR and pS6 levels.

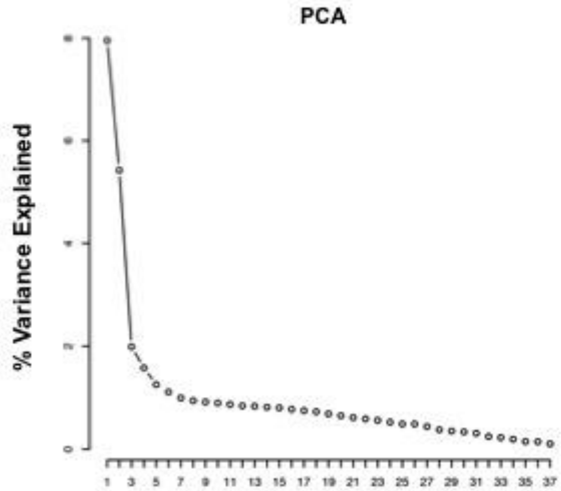

Supplementary Figure 4. Principal Component Loadings

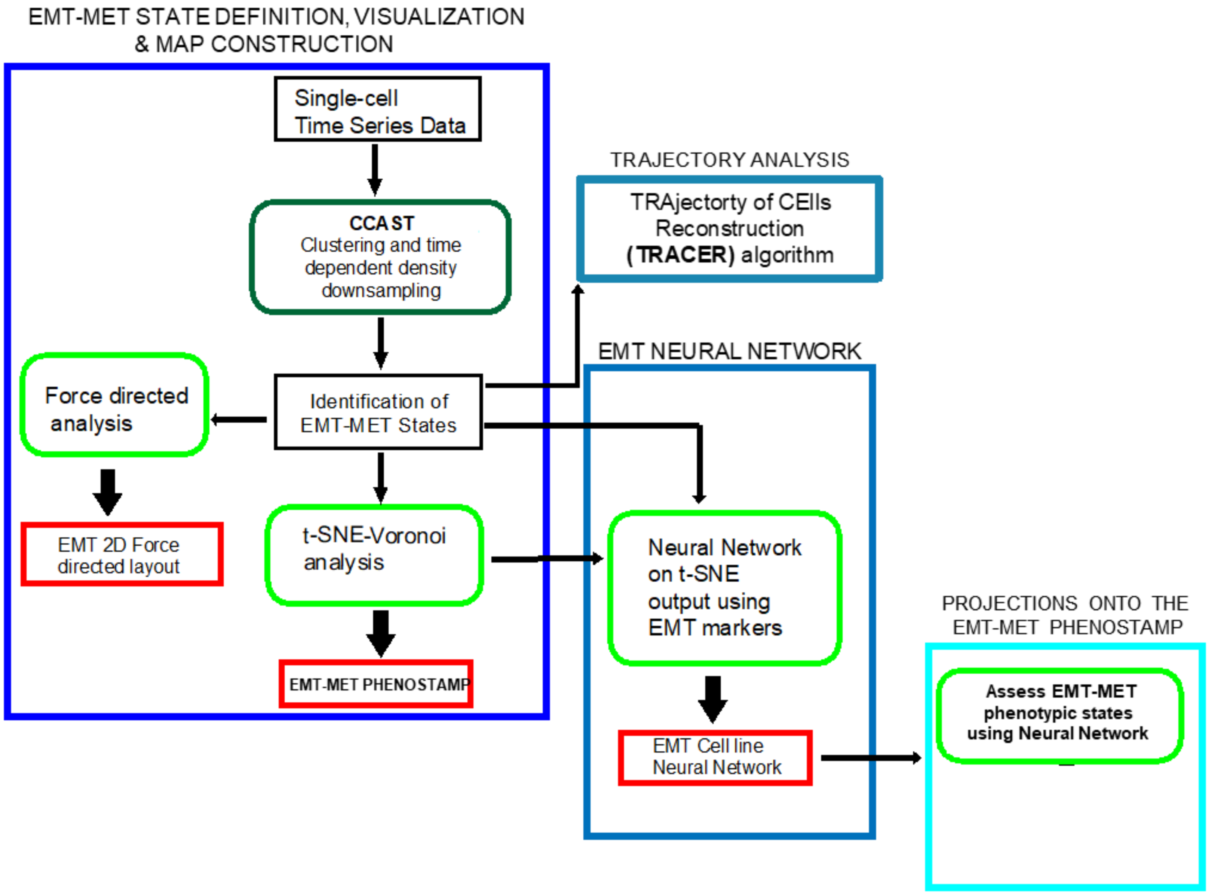

Supplementary Figure 5. Flowchart depicting computational analyses and tools applied and/or developed for mapping EMT-MET states and trajectories. Related to Figures 3-6. See Methods for details

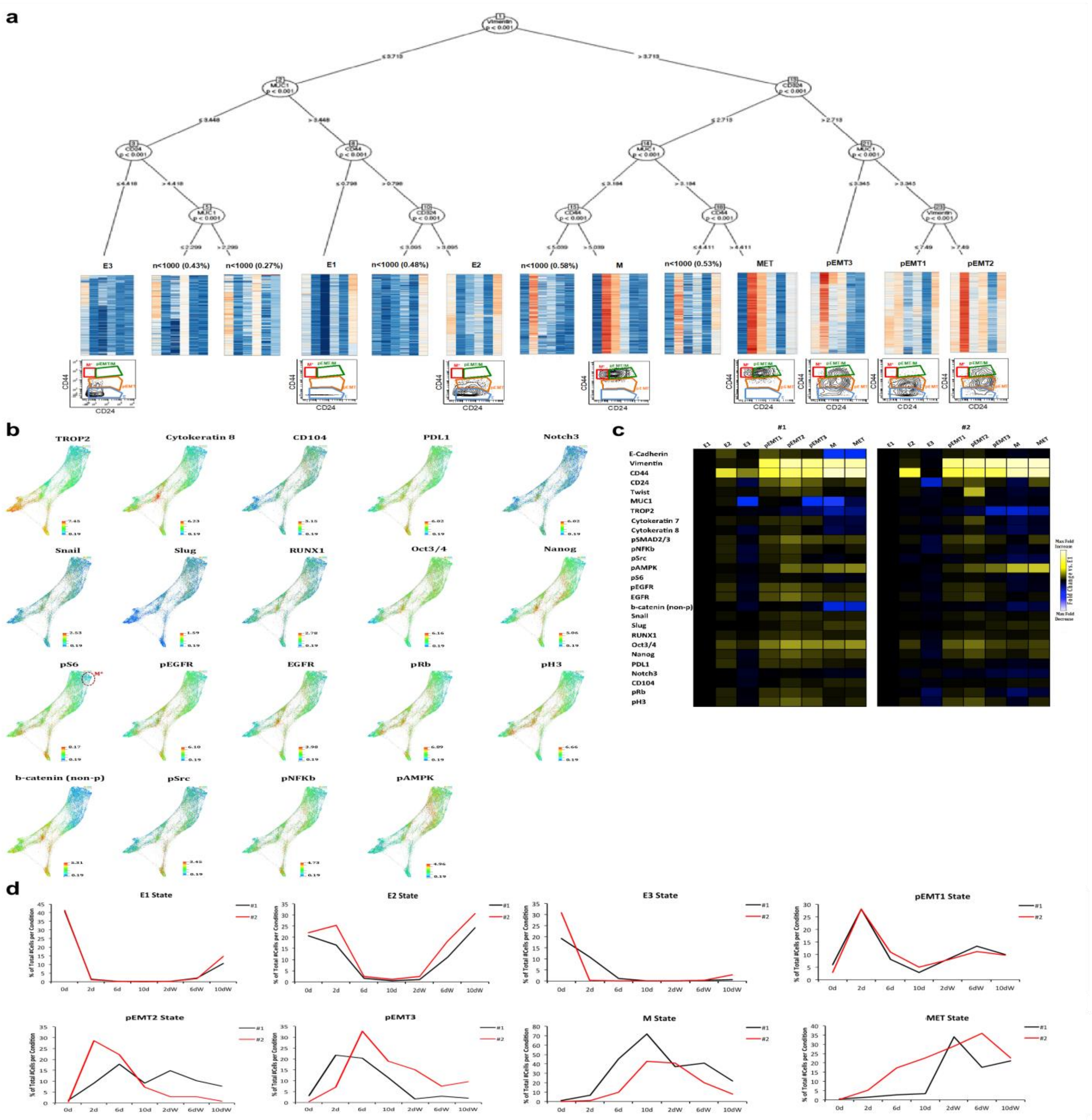

Supplementary Figure 6. CCAST identifies 8 computationally-derived EMT and MET states. Related to Figure 3. **(a)** CCAST decision tree for the HCC827 mass cytometry data derived from all 28 markers showing 13 distinct subpopulations using 6 clustering markers: E-Cadherin, Vimentin, CD44, CD24, MUC1 and Twist. Eight of the 13 clusters met our criteria (n>1000 cells, ≥1% of pooled cells) for downstream analysis. (*Bottom*) Heat maps of normalized data from all subgroups derived from the decision tree above and their expression for the aforementioned 6 clustering markers. Below each computationally derived EMT-MET state is the respective CD44/CD24 plot showing how it relates to the

EMT canonical states. **(b)** Force-directed layouts (FDLs) colored by protein expression levels of the remaining markers analyzed with mass cytometry. Note red circled area on the pS6 FDL, indicating M^*, $CD44^{hi}/CD24^{lo}$ subpopulation of cells. **(c)** Heat map summary depicting fold change expression of each marker per EMT/MET state towards E1 state from experiment shown in Figure 3 (#1) and in an independent biological replicate experiment (#2). **(d)** EMT/MET state dynamics shown for experiment in Figure 3B (black lines, #1) alongside matched states in an independent biological replicate experiment (red lines, #2)

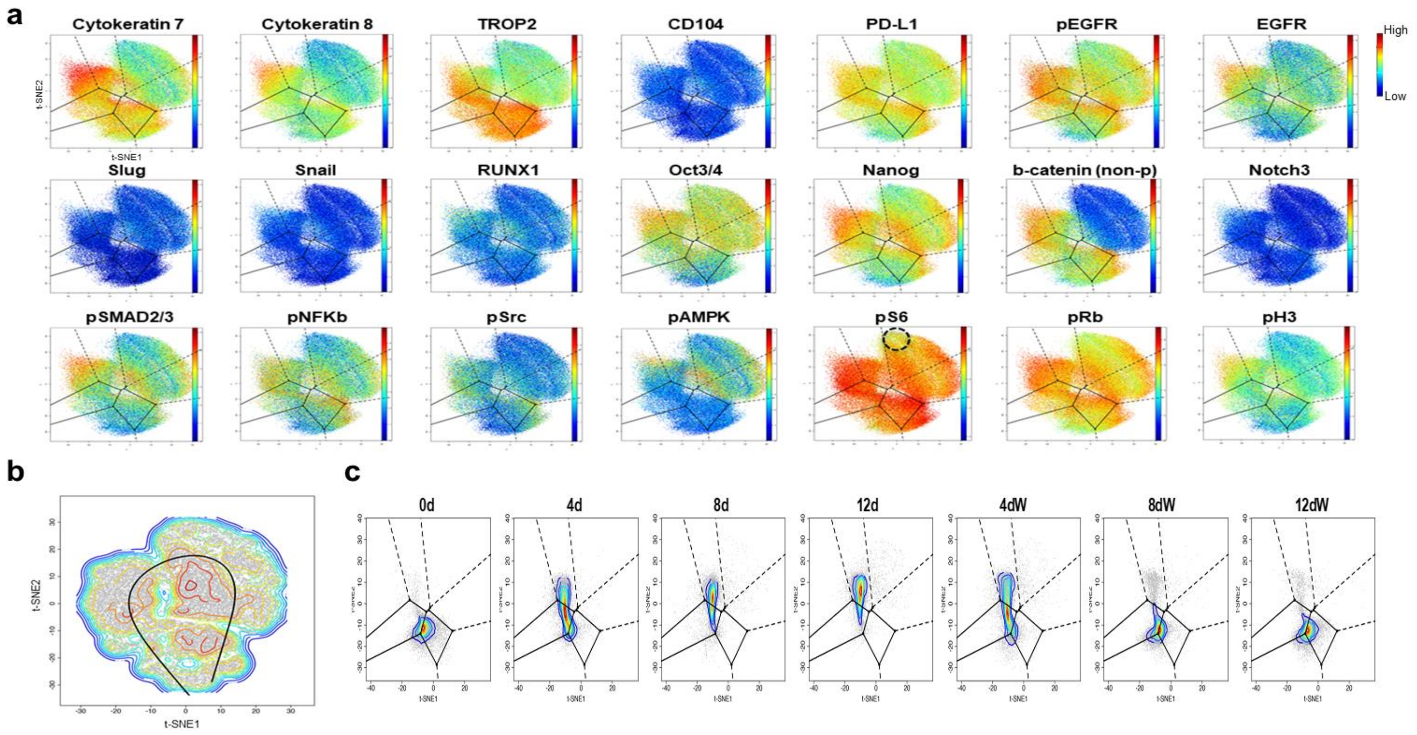

Supplementary Figure 7. Construction of an EMT-MET PHENOtypic STAte MaP (PHENOSTAMP). Related to Figure 4. **(a)** Expression profiles of remaining markers in pooled HCC827 time-point data visualized on the EMT-MET state map. Circled areas on pS6 plot depicts the M*, CD44^{hi}/CD24^{lo} subpopulation of cells. **(b)** Slingshot analysis confirms the trajectory that involves the MET state. MET is visited by cells during TGFβ withdrawal conditions and is unique to one of two possible MET scenarios. **(c)** Time-point t-SNE density plots of HCC827 cells that did not efficiently undergo EMT during a TGFβ time-course experiment. Note that at all time-points a very small number of cells occupy the M region and almost no cells occupy the MET region of the map.

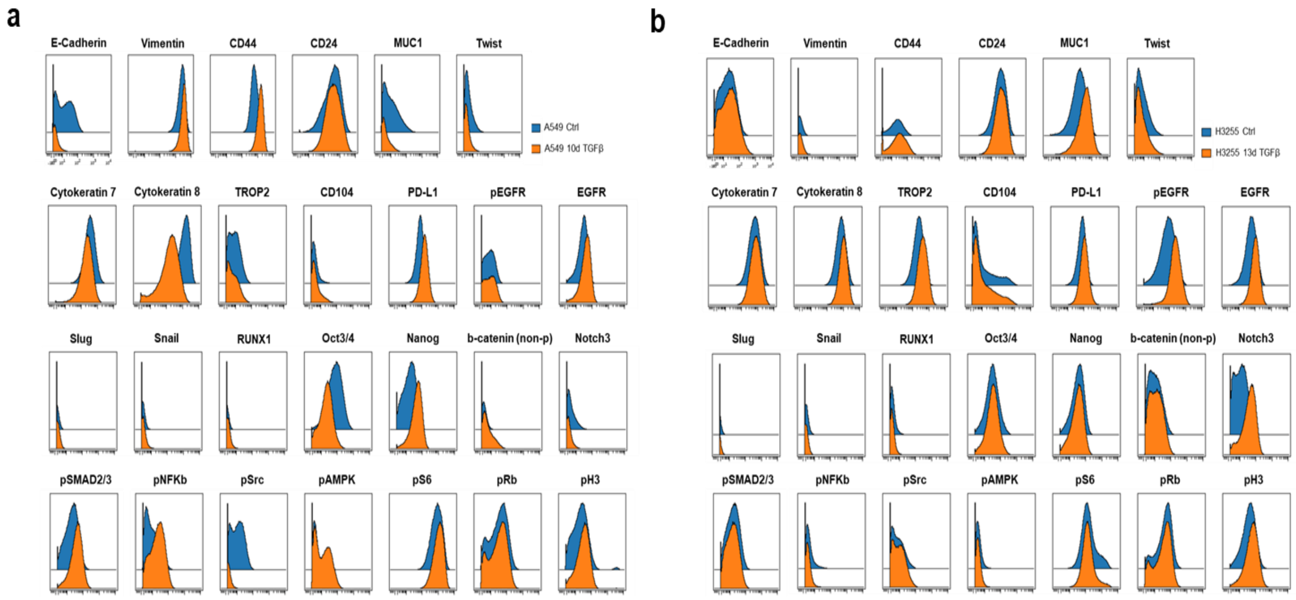

Supplementary Figure 8. Expression profiles of all cellular markers analyzed with mass cytometry in **(a)** A549 and **(b)** H3255 cell line samples that were subsequently projected on the EMT-MET PHENOSTAMP. Related to Figure 5

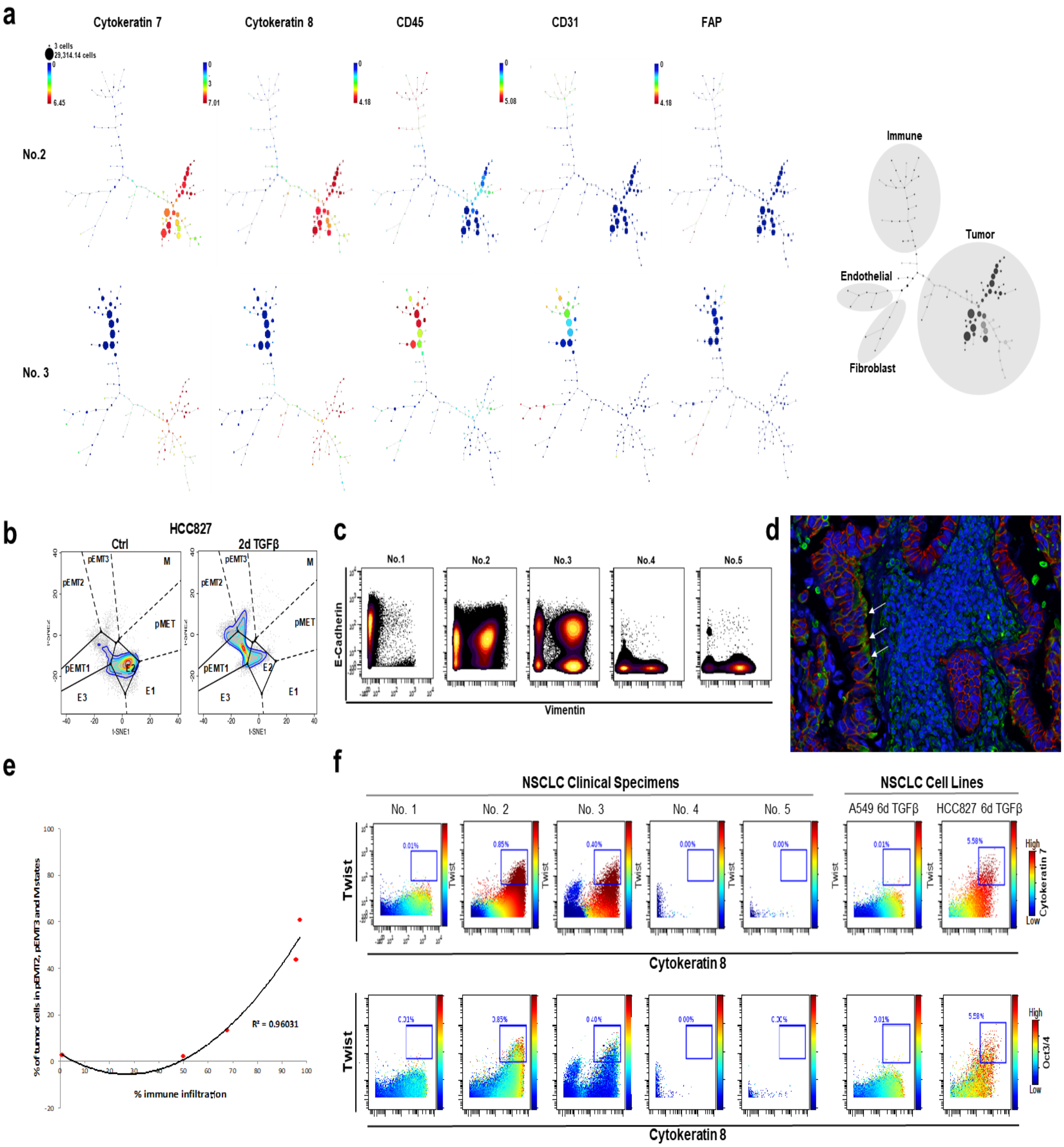

Supplementary Figure 9. Mass cytometry analysis and projection of NSCLC clinical specimens onto the EMT-MET PHENOSTAMP. Related to Figure 6. **(a)** SPADE analysis showing efficient *in silico* separation of tumor cells with the use of CD45, CD31 and FAP antibodies in clinical specimens No. 2 and 3. Cytokeratin 7 and 8 expression profiles are shown to confirm absence in immune, endothelial and stromal populations, and variable expression in tumor cells. Staining profiles of all clustering markers (CD45, CD31, FAP, Cytokeratins 7 and 8) can be used to identify and gate out cell populations in clinical

specimens analyzed with mass cytometry (SPADE illustration of clinical specimen No. 2 to the right, shaded areas. Down-sampled events target: 10 %, number of nodes target: 120. **(b)** Projection of HCC827 cells (ctrl, 2-day TGFβ) that were stained and analyzed alongside clinical specimens for validation purposes. **(c)** E-Cadherin/Vimentin mass cytometry plots of the 5 NSCLC clinical specimens that were analyzed. **(d)** Immunofluorescent staining of matched tissue from specimen No. 2. Arrows indicate pEMT cells co-expressing E-Cadherin (red) and Vimentin (green). **(e)** % of tumor cells projected on pEMT2, pEMT3 and M areas of the EMT-MAP map vs. % immune infiltration shows positive correlation between the two in the five NSCLC clinical specimens analyzed. **(f)** Twist^{+}/Cytokeratin 7^{+}/Cytokeratin 8^{+}/Oct3/4^{+} subpopulation of cells is detected in EGFR mutated NSCLC (Clinical Specimens No. 2 and 3).

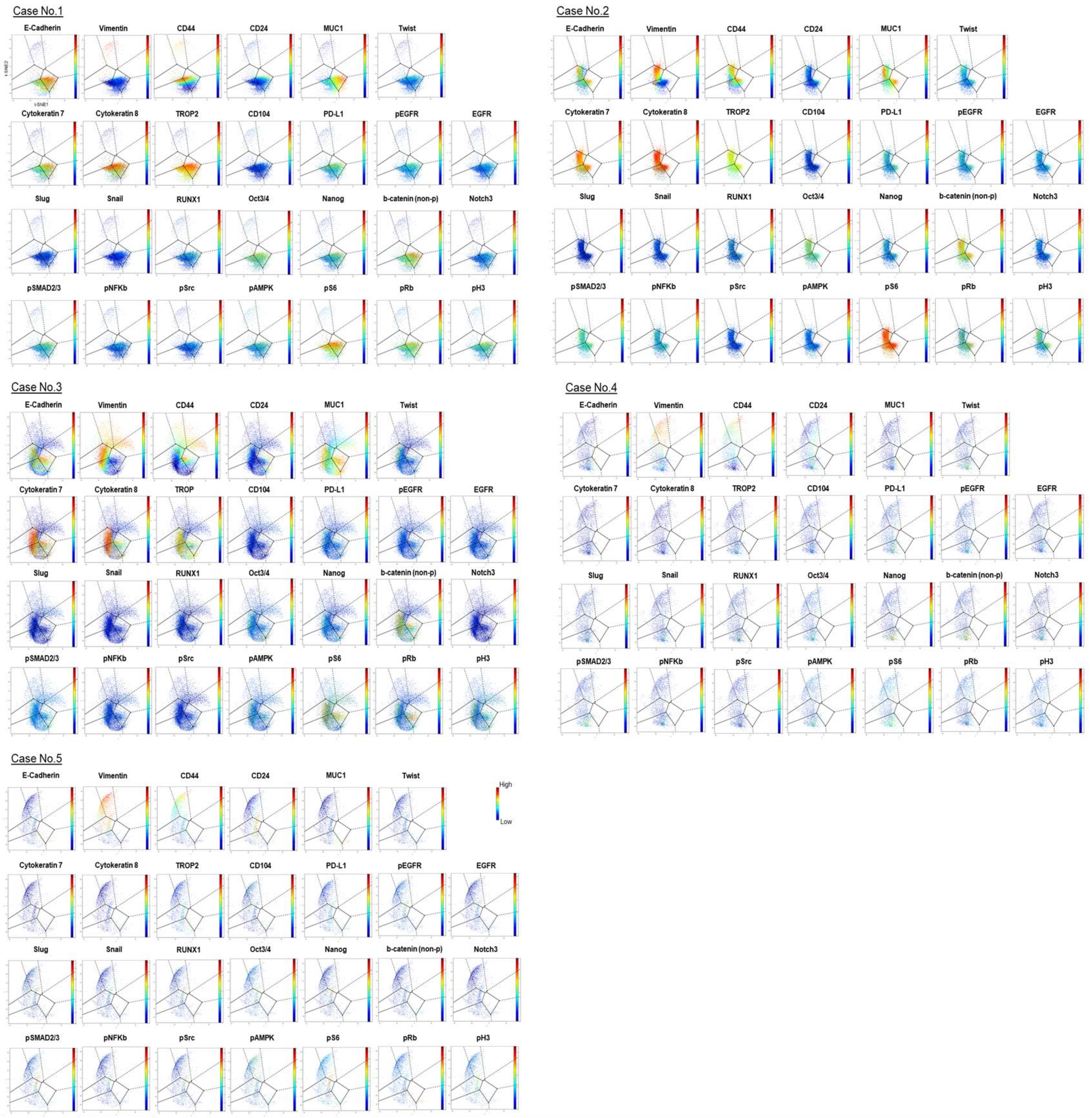

Supplementary Figure 10. Expression profiles of all markers in the NSCLC clinical specimens visualized on the EMT-MET PHENOSTAMP. Related to Figure 6. Clinical specimens No.1, 2, and 3 all share EGFR mutations and map primarily on E and pEMT1 regions of the map, with the exception of specimen No.3 which carries an additional TP53 mutation, and has cells mapping on more mesenchymal regions of the map including those of pEMT2, pEMT3, M and MET. Clinical specimens No. 4 and 5 carry mutations other than EGFR, specifically TP53 and KRAS respectively, and have a proportion of cells mapping on the mesenchymal pEMT2 and M regions of the map. Of note, specimens No. 4 and 5 had the highest % immune infiltration at 95.85% and 97.20% respectively.
