# Supplementary material for: Mapping lung cancer epithelial-mesenchymal transition states and trajectories with single-cell resolution
Source: Nat Commun. 2019 Dec 6;10:5587. doi: 10.1038/s41467-019-13441-6 (PMC6898514; doi:10.1038/s41467-019-13441-6)
Supplement: Supplementary file 3 — Reporting Summary [file 41467_2019_13441_MOESM3_ESM.pdf]

nature research

Corresponding author(s): Dr. Sylvia K Plevritis

Last updated by author(s): Oct 9, 2019

Reporting Summary

Nature Research wishes to improve the reproducibility of the work that we publish. This form provides structure for consistency and transparency in reporting. For further information on Nature Research policies, see Authors & Referees and the Editorial Policy Checklist.

Statistics

For all statistical analyses, confirm that the following items are present in the figure legend, table legend, main text, or Methods section.

n/a	Confirmed	
☐	☒	The exact sample size (*n*) for each experimental group/condition, given as a discrete number and unit of measurement
☐	☒	A statement on whether measurements were taken from distinct samples or whether the same sample was measured repeatedly
☐	☒	The statistical test(s) used AND whether they are one- or two-sided<br>*Only common tests should be described solely by name; describe more complex techniques in the Methods section.*
☐	☒	A description of all covariates tested
☐	☒	A description of any assumptions or corrections, such as tests of normality and adjustment for multiple comparisons
☐	☒	A full description of the statistical parameters including central tendency (e.g. means) or other basic estimates (e.g. regression coefficient) AND variation (e.g. standard deviation) or associated estimates of uncertainty (e.g. confidence intervals)
☒	☐	For null hypothesis testing, the test statistic (e.g. *F*, *t*, *r*) with confidence intervals, effect sizes, degrees of freedom and *P* value noted<br>*Give P values as exact values whenever suitable.*
☒	☐	For Bayesian analysis, information on the choice of priors and Markov chain Monte Carlo settings
☐	☒	For hierarchical and complex designs, identification of the appropriate level for tests and full reporting of outcomes
☒	☐	Estimates of effect sizes (e.g. Cohen's *d*, Pearson's *r*), indicating how they were calculated

Our web collection on statistics for biologists contains articles on many of the points above.

Software and code

Policy information about availability of computer code

Data collection	N/A
Data analysis	Custom algorithms PHENOSTAMP [https://github.com/anchangben/PHENOSTAMP] and TRACER [https://github.com/nignatiadis/TRACER] were developed for data analysis in this study and have been made available on GitHub. In addition, published tools were also used in data analysis, specifically CCAST for clustering and Vortex for force-directed layout visualization of single-cell data, with the corresponding references provided.

For manuscripts utilizing custom algorithms or software that are central to the research but not yet described in published literature, software must be made available to editors/reviewers. We strongly encourage code deposition in a community repository (e.g. GitHub). See the Nature Research guidelines for submitting code & software for further information.

Data

Policy information about availability of data

All manuscripts must include a data availability statement. This statement should provide the following information, where applicable:

- Accession codes, unique identifiers, or web links for publicly available datasets
- A list of figures that have associated raw data
- A description of any restrictions on data availability

The mass cytometry time-series data have been deposited in the Cytobank Stanford database under the name “Karacosta et al. HCC827 EMT time-series mass cytometry data” [https://stanford.cytobank.org/cytobank/experiments/26555]. All the other data supporting the findings of this study are available within the article and its supplementary information files and from the corresponding author upon reasonable request. The source data underlying Figs. 1a, 1e, 3b, 3c, 4e and Supplementary Fig. 1g are provided as a Source Data file and these include all raw single-cell data resulting from the CCAST analysis described in the paper.

October 2018

Field-specific reporting

Please select the one below that is the best fit for your research. If you are not sure, read the appropriate sections before making your selection.

☒ Life sciences ☐ Behavioural & social sciences ☐ Ecological, evolutionary & environmental sciences

For a reference copy of the document with all sections, see nature.com/documents/nr-reporting-summary-flat.pdf

Life sciences study design

All studies must disclose on these points even when the disclosure is negative.

Sample size	N/A
Data exclusions	N/A
Replication	The in vitro time series experiment and mass cytometry analysis was repeated in an independent biological replicate, to demonstrate reproducibility; this replicate is described in the Supplementary information. In addition, replicates of HCC827 experimental conditions were independently ran and analyzed with mass cytometry each time a projection (of other cell lines and clinical samples) was performed on the EMT-MET PHENOSTAMP - this is described in the main paper and respective figure legends.
Randomization	N/A
Blinding	N/A

Reporting for specific materials, systems and methods

We require information from authors about some types of materials, experimental systems and methods used in many studies. Here, indicate whether each material, system or method listed is relevant to your study. If you are not sure if a list item applies to your research, read the appropriate section before selecting a response.

Materials & experimental systems

n/a	Involved in the study	
☐	☒	Antibodies
☐	☒	Eukaryotic cell lines
☒	☐	Palaeontology
☒	☐	Animals and other organisms
☐	☒	Human research participants
☐	☒	Clinical data

Methods

n/a	Involved in the study	
☒	☐	ChIP-seq
☐	☒	Flow cytometry
☒	☐	MRI-based neuroimaging

Antibodies

Antibodies used

Flow Cytometry fluorophore conjugated primary antibodies used: PE/Cy7 E-Cadherin (Biolegend, Clone 67A4, #324115, Lot# B240261), Alexa 488 Vimentin (BD, Clone RV202, #562338, Lot# 6252777), Pacific Blue CD44 (Biolegend, Clone IM7, #103019, Lot# B182715), APC/Cy7 CD24 (Biolegend, Clone ML5, #311131, Lot# B210741), Alexa 647 Twist (Bioss, #bs-2441R, Lot# AE084712).

Confocal Imaging fluorophore conjugated primary antibodies: Alexa 488 Vimentin Antibody (BD, Clone RV202, #562338, Lot# 6252777) and Alexa 647 E-Cadherin Antibody (BD, Clone 67A4 # 324112, Lot# B197755)

Immunoblot antibodies: E-Cadherin (BD, #610181, Lot# 2300996, 1:5,000), Vimentin (Abcam, ab92547, Lot# GR219216-17 1:500), CD44 (CST, #3570, Lot# 10, 1:200), Zeb (CST, #3396, Lot# 6, 1:200), Slug (CST, #9585, Lot# 3, 1:200), Twist (GeneTex, #GTX127310, Lot# 41220, 1:500), GAPDH (CST, #5174, Lot# 6, 1:10,000)

A detailed table of all Mass Cytometry antibodies (custom ordered BSA/carrier free and metal conjugated) is provided in Supplementary Information (Clone, Vendor, Metal, Mass, Final Concentration) and additional information is provided in Methods. Here we provide in addition, catalogue # and specific phospho sites for the majority of antibodies used:
CD45 (Biolegend, #3089003B), CD44 (Biolegend, #103051), pSrc (BD, phospho-site Y418), pEGFR (CST, #3777, phospho-site Tyr1068), EGFR (CST, #4267), TROP2 (R&D, # MAB650), Oct3/4 (BD, #561555), PD-L1 (Biolegend, # 329702), Snail (CST, #3879BF), Nanog (CST, #3580), p-H3 (Biolegend, phospho-site S28), CD24 (Biolegend #311102), p-SMAD2/3 (CST, #8828BF, phospho-sites S465/467, S423/425), p-NFkb (BD, phospho-site S529), p-S6 (BD, #624084, phospho-site S235/236), phospho-Rb (BD, #558389, phospho-site S807/811), Twist (Bioss, polyclonal, #bs-2441R), Non-phospho-b-catenin (CST, #8814BF), CD31 (Biolegend, #303102), CD104 (Fluidigm, #3173008B), Vimentin (CST, #5741BF), p-AMPK (CST, #2535BF, phospho-site T172)

Validation	All flow cytometry and confocal imaging antibodies (except for Twist antibody) have been quality tested for flow cytometry per manufacture's website (Biolegend and BD) and reactive with human. In most cases recommended concentrations were used, except in the cases where additional titration was necessary.<br>All immunoblotting antibodies per respective manufacture's website are tested and applicable for Western blotting and reactive to human.<br>All mass cytometry antibodies per manufacture's website are reactive with human and were titrated. Optimized concentrations per antibody used are provided in Supplementary Table 1.<br><br>Additional notes: EMT Induction in cell culture, served as an additional control towards validating the main/canonical EMT marker antibodies (E-Cadherin, Vimentin, CD44, CD24). Our NSCLC cell lines, are reported in the literature as epithelial cells and this is evident by the marker expression in all applications used in the paper (immunoblotting, flow cytometry and mass cytometry). In addition, the main EMT marker expression is agreeable between flow cytometry and mass cytometry in our cell line experiments.<br><br>Low endogenous EMT transcription factor expression (as well as overall decrease of Twist expression in mesenchymal cells - induced by TGFb treatment) observed in mass cytometry experiments is agreeable with Western Blot applicable antibodies and this is provided in Supplementary Information.

Eukaryotic cell lines

Policy information about cell lines

Cell line source(s)	All 3 NSCLC cell lines were a generous gift from a Stanford collaborator, Dr. Parag Mallick
Authentication	Cell lines were not authenticated
Mycoplasma contamination	Cell lines were not tested for Mycoplasma contamination
Commonly misidentified lines<br>(See ICLAC register)	N/A

Human research participants

Policy information about studies involving human research participants

Population characteristics	The target patient population (5 patients in total for this study) consisted of individuals (older than 18 years) undergoing a surgical procedure for lung adenocarcinomas in the thoracic cavity requiring diagnosis and/or surgical removal with no previous treatment given.
Recruitment	Typically, patients age 18-65 were approached for recruitment. Also, patients older than 65 were approached for recruitment since a significant number of people who develop lung cancer are older than 65. Both males and females of varying ethnicity were asked to participate. All patients with the suspected or an established diagnosis of lung cancer at Stanford Hospital and Clinics, Stanford Cancer Center, or Palo Alto VAHCS were eligible. Patients undergoing surgery for thoracic pathology at these institutions were asked to voluntarily donate tissues remaining after pathologic diagnosis and informed consent was given.
Ethics oversight	Stanford University Institutional Review Board

Note that full information on the approval of the study protocol must also be provided in the manuscript.

Clinical data

Policy information about clinical studies

All manuscripts should comply with the ICMJE guidelines for publication of clinical research and a completed CONSORT checklist must be included with all submissions.

Clinical trial registration	N/A. This was not a clinical trial but a tissue collection human protocol
Study protocol	Protocol # 15166 titled "Donating Tissue for Thoracic Surgery Research", of which Dr. Joseph B. Shrager is Director, can be provided as a PDF upon request.
Data collection	Patients consented at the Stanford Thoracic Surgery Clinic between 01/01/2017-5/10/18 and samples were collected for mass cytometry analysis.
Outcomes	N/A

Flow Cytometry

Plots

Confirm that:

☒ The axis labels state the marker and fluorochrome used (e.g. CD4-FITC).

☒ The axis scales are clearly visible. Include numbers along axes only for bottom left plot of group (a 'group' is an analysis of identical markers).

☒ All plots are contour plots with outliers or pseudocolor plots.

☒ A numerical value for number of cells or percentage (with statistics) is provided.

Methodology

Sample preparation	Note: Most data and conclusions presented in the paper result from mass cytometry analysis (which is described in detail in the Methods section). Flow Cytometry was used for only the data presented in Figure 1, and Supplementary Figure 1 and 2. Concerning the first box, markers are stated on axis, but not the fluorochrome. This information can be found in the Methods section as well as in the Antibodies section above.<br>Flow Cytometry Sample (cell line only) preparation: Following treatments cells were lifted off tissue culture plates using TrypLE (Life technologies, #12605-010). After counting and assessing % viability with Trypan Blue exclusion, cell aliquots (1 million cells) per condition were fixed by adding PFA at a final concentration of 1.6% for 10 minutes at room temperature. Cells were then centrifuged at 500g for 5 minutes at 4 degrees Celsius to pellet cells and remove PFA and washed once with cell staining media (CSM, 0.5% w/v BSA, 0.02% w/v NaN3 in PBS). Cells were permeabilized with methanol solution for 10 minutes on ice and optionally stored at -80 degrees Celsius for long-term storage. After two washes with CSM, master mix of antibodies was added to pelleted cells at a total volume of 100uL for 30 minutes in the dark at room temperature. Following two washes with CSM, cells were analyzed.
Instrument	LSR II.UV at Stanford Shared FACS facility (https://facs.stanford.edu/)
Software	Data were analyzed using Cytobank (www.cytobank.org)
Cell population abundance	No sorting was performed
Gating strategy	FSC-A/FSC-H gating was used to gate singlets and dead cells were gated out using the LIVE/DEAD Fixable Blue Dead Cell Stain Kit (Life Technologies, #L23105) as per manufacturer's instructions. Boundaries between "positive" and "negative" staining were defined with a combination of "No antibody" and FMO control staining conditions as well as differences between EMT induction experimental conditions compared to control experimental conditions.

☒ Tick this box to confirm that a figure exemplifying the gating strategy is provided in the Supplementary Information.
